# Effect of Nuts on Markers of Inflammation and Oxidative Stress: A Narrative Review

**DOI:** 10.3390/nu15051099

**Published:** 2023-02-22

**Authors:** Sujatha Rajaram, Nagila Raquel Teixeira Damasceno, Ribanna Aparecida Marques Braga, Raquel Martinez, Penny Kris-Etherton, Aleix Sala-Vila

**Affiliations:** 1 School of Public Health, Loma Linda University, Loma Linda, CA 92354, USA; 2 Department of Nutrition, School of Public Health, University of São Paulo, São Paulo 05508-220, Brazil; 3 Department of Nutritional Sciences, Pennsylvania State University, State College, PA 16801, USA; 4 Cardiovascular Epidemiology and Genetics Group, IMIM Hospital del Mar Medical Research Institute, 08003 Barcelona, Spain

**Keywords:** inflammation, non-communicable diseases, oxidative stress, peanuts, tree nuts

## Abstract

Oxidative stress and inflammation are mediators in the pathophysiology of several non-communicable diseases (NCDs). Tree nuts and peanuts lower risk factors of cardiometabolic disease, including blood lipids, blood pressure and insulin resistance, among others. Given their strong antioxidant/anti-inflammatory potential, it is plausible that nuts may also exert a favorable effect on inflammation and oxidative stress. Evidence from systematic reviews and meta-analyses of cohort studies and randomized controlled trials (RCTs) suggest a modest protective effect of total nuts; however, the evidence is inconsistent for specific nut types. In this narrative review, the state of evidence to date is summarized for the effect of nut intake on biomarkers of inflammation and oxidative stress, and an attempt is made to define the gaps in research while providing a framework for future research. Overall, it appears that some nuts, such as almonds and walnuts, may favorably modify inflammation, and others, such as Brazil nuts, may favorably influence oxidative stress. There is a pressing need for large RCTs with an adequate sample size that consider different nut types, and the dose and duration of nut intervention, while evaluating a robust set of biomarkers for inflammation and oxidative stress. Building a stronger evidence base is important, especially since oxidative stress and inflammation are mediators of many NCDs and can benefit both personalized and public health nutrition.

## 1. Introduction

Non-communicable diseases (NCDs) are related to lifestyle factors including smoking, sedentary habits, and an unhealthy dietary pattern [1]. Oxidative stress and low-grade chronic inflammation are common mediators in the pathophysiology of many age-related NCDs, including obesity, cardiovascular disease (CVD), type-2 diabetes (T2D), some cancers, and neurodegenerative diseases [2]. Diet and lifestyle modification strategies are cornerstones for preventing NCDs. The evidence supports the role of plant-based diet patterns and plant foods such as tree nuts and peanuts in preventing NCDs and comorbidities, thereby favorably modifying the incidence and mortality of these diseases [3,4,5,6,7]. 

**Figure 1:**
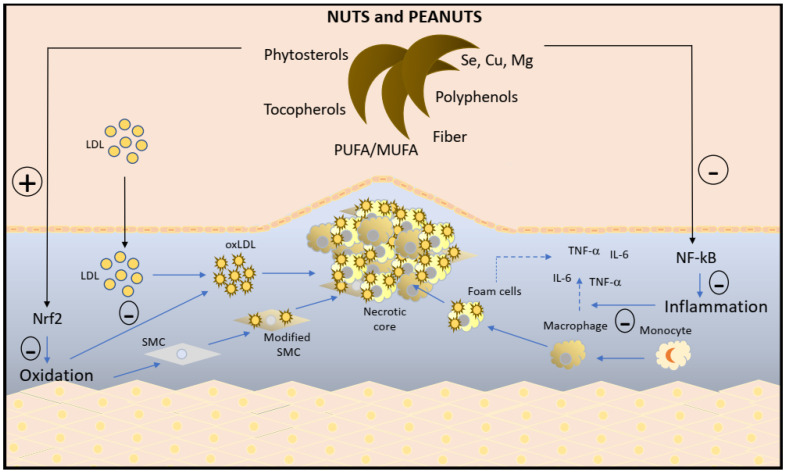
Inflammatory and oxidative processes modulated by nutrients and bioactive substances in tree nuts and peanuts. Fiber, phytosterols, PUFA/MUFA and mineral antioxidants such as Se, Cu, and Mg in tree nuts and peanuts can improve lipid metabolism through a cholesterol-lowering effect on low-density lipoprotein (LDL). Additionally, antioxidants (polyphenols and tocopherols) quench free radicals, avoiding the oxidative modification of LDL (oxLDL). Together, this process prevents monocyte migration and macrophage differentiation, and the consequent endocytosis of oxLDL by macrophages and the formation of foam cells. Increased levels of free radicals in the sub endothelial space stimulate changes in the smooth muscle cell (SMC) phenotype, favoring uptake of oxLDL. This complex process is essential to induce and maintain the low-grade inflammation characterized by the continuous synthesis of inflammatory cytokines (TNF-α, IL-6, and other markers) common in atherosclerosis and present in many other chronic diseases. Tree nuts and peanuts rich in bioactive substances can modulate multiple inflammation and oxidation pathways. Additionally, nut antioxidants can directly quench reactive oxygen species produced in the cell, reduce oxidative stress, and suppress NFκβ expression and downstream pro-inflammatory cytokine generation. Nut antioxidants are cofactors of several antioxidant enzymes. They can increase their activity, upregulate the gene expression of Nrf2, and increase the expression of antioxidant response element gene expression.

Reactive oxygen species (ROS) and nitrogen oxidative species (NOS) are part of the normal cellular processes, but the oxidative stress induced by the imbalance of antioxidant systems triggers chronic low-grade inflammation (Figure 1) that contributes to the development of atherosclerosis, CVD, and insulin resistance-related diseases [2,8,9]. In the setting of oxidative stress there is the activation of the regulatory transcription factor, nuclear factor kappa beta (NFκβ), resulting in the release of pro-inflammatory cytokines and the inhibition of nuclear factor-erythroid factor 2-related factor 2 (Nrf2), all leading to more inflammation [10,11]. Plant foods like nuts are rich in phytochemicals and other antioxidant nutrients, with a potential to counteract oxidative stress and inflammation. Tree nuts and peanuts lower major risk factors of cardiometabolic disease, including blood lipids/lipoproteins, blood pressure, endothelial dysfunction and insulin resistance [12,13,14,15]. Given their strong antioxidant/anti-inflammatory potential, nuts may also exert a favorable effect on other risk factors of cardiometabolic disease, such as inflammation and oxidative stress. 

Tree nuts (almonds, Brazil nuts, cashew, hazelnuts, macadamia, pecans, pine nuts, pistachios, and walnuts) and peanuts (classified as a legume but considered a nut due to its similar nutrient profile and health benefits) are plant foods that offer a unique combination of macronutrients, micronutrients, and phytonutrients. They are rich in unsaturated fat, which includes polyunsaturated fat (PUFA), both linoleic and α-linolenic acid (ALA), and monounsaturated fat (MUFA). Walnuts and pine nuts have a significant amount of PUFAs, while all nuts are high in MUFAs. Nuts also have a plethora of powerful antioxidant and anti-inflammatory bioactives, including tocopherols, selenium, zinc, magnesium, fiber, phytosterols, and polyphenols [16,17,18,19]. Many of these nutrients and non-nutrients present in nuts exert antioxidant and anti-inflammatory effects independently [18,19], but their presence in a whole food matrix may promote synergistic effects. 

Early nut intervention trials primarily observed a lipid-lowering effect of nuts; interestingly, the CVD risk reduction was greater than predicted based on low-density lipoprotein cholesterol (LDL-C) lowering alone [12,13,14,19,20]. To gain a deeper understanding of the other mechanisms beyond blood lipids that might contribute to the overall CVD risk reduction, the effect of nuts on biomarkers of inflammation and oxidative stress were evaluated mostly as secondary outcomes. This narrative review summarizes the current understanding of the role of tree nuts and peanuts on oxidative stress and inflammation biomarkers, identifies the gaps in this area, and provides a framework for future research. This review was presented at an international conference, “NUTS 2022, Where we are and where we are going in research”, in the session on “nuts, inflammation and oxidation”. While not exhaustive, this review has considered all relevant systematic reviews, cohort studies and randomized controlled trials (RCTs) published to date. 

## 2. Nuts and Inflammation and Oxidative Stress

This section explores evidence from cohort studies and RCTs on the association or effect, respectively, of the various tree nuts and peanuts on markers of inflammation and oxidative stress. Serum markers of inflammation frequently assayed include *C*-reactive protein (CRP), tumor-necrosis factor-α (TNF-α), interleukin-6 (IL-6) and interleukin-1β (IL-1β), as well as adhesion molecules E-selectin, intercellular (ICAM-1), and vascular cell adhesion (VCAM-1) molecules. Enzymes play an integral part in managing antioxidant and oxidant homeostasis, from which superoxide dismutase (SOD) converts superoxide into hydrogen peroxide and dioxygen [21], while glutathione S-transferase (GST) participates in the phase II detoxification process [22]. The markers of oxidative stress frequently assessed in studies are oxidized LDL (oxLDL), lipid peroxides malondialdehyde (MDA), DNA damage marker, 8-hydroxy deoxy guanine (8-OHdG), antioxidant enzyme activity, and antioxidant capacity. The evidence to date from cohort studies and RCTs is presented below. 

### 2.1. Evidence from Cohort Studies

Systematic reviews and meta-analyses of prospective cohort studies investigated the association between nut consumption and coronary heart disease (CHD) risk, stroke, hypertension, and T2D [5,6,23,24,25]. Irrespective of follow-up time (3.8 to 26 years), geographical regions, and variation in cohort size (175,000 to >300,000), higher nut intake was inversely associated with CHD risk (relative risk [RR] = 0.81; 95% CI = 0.72–0.91, heterogeneity [*I*^2^] = 56.8%, *p* = 0.018) [6]. In cohort studies of hypertension cases versus control, a marginal association between higher nut intake and reduced risk of hypertension was noted (RR = 0.66; 95% CI = 0.44–1.00, *I*^2^ = 75.9%, *p* = 0.006) but no association was observed for T2D or stroke risk. With respect to nut consumption and colorectal cancer risk, the pooled relative risk for the highest versus lowest (never) categories of nut consumption was 0.91 (95% CI = 0.79–1.05, *I*^2^ = 49.1%, *p* = 0.08). In case–control studies, there was a significant reduction in colorectal cancer risk (RR = 0.84; 95%CI = 0.71–0.99) with nut consumption [6]. 

The association of nuts and peanuts on the incidence of total mortality, mortality related to CVD, cancer and all-cause among T2D subjects was evaluated in a cross-sectional sample from the Nurses’ Health Study (NHS) (1980–2014) and Health Professionals Follow-Up Study (HPFS) (1986–2014) cohorts including 16,217 men and women with T2D at baseline or diagnosed during follow-up [24]. Nuts were associated with a significant reduction in the incidence of total CVD (hazard ratio [HR] = 0.80; 95% CI = 0.70–0.92), and CHD (HR = 0.77; 95% CI = 0.65–0.79), and CVD-specific mortality (HR = 0.61; 95% CI = 0.49–0.79), cancer mortality (HR = 0.73; 95% CI = 0.60–0.90) and all-cause mortality (HR = 0.67; 95% CI = 0.60–0.74). However, peanuts were associated only with a decrease in all-cause mortality. Collectively, the cohort studies, which have considered many relevant covariates (age, BMI, physical activity, smoking, alcohol, energy intake) in their statistical models, confirmed the positive relationship of nuts and peanuts on mortality incidence; however, neither oxidative stress nor inflammation outcomes were considered. 

The cohort studies that have determined the association of nuts and peanut intake on inflammatory markers and their role in risk, incidence, and mortality from NCDs are presented in Table 1. Li et al. [25] examined the association between nut consumption and incident CVD in a subsample from the NHS composed of 6309 women with T2D. From a semi-quantitative food frequency questionnaire (FFQ), habitual nut consumption was grouped into four categories based on the number of servings (serving size, 28 g (1 ounce) for nuts and 16 g for peanut butter): mostly never, 1–3 servings/month to 1 serving/week, 2–4 servings/week, or ≥5 servings/week. Although a higher consumption of nuts and peanuts was associated with lower CVD risk in women with T2D, it was not significantly associated with the inflammatory markers, including tumor necrosis factor receptor 2 (TNFR), ICAM-1, E-selectin, CRP, or fibrinogen. A cross-sectional study from the NHS cohort including 987 women with diabetes assessed the adherence level to the Mediterranean dietary (MedDiet) pattern on a 9-point scale. The higher (6–9 score) adherence level to the MedDiet was associated with higher adiponectin level in comparison to individuals in the lower category of adherence (0–3 score), independent of age, total energy intake, BMI, waist circumference, physical activity, and smoking status. To investigate which food groups in the MedDiet were able to explain the improvement in adiponectin level, using an age and energy-adjusted model, women with diabetes in the highest quintile of nut consumption had adiponectin levels significantly higher, by 23%, than those in the lowest quintile, and this remained significant even when adjusted for BMI, smoking status, activity level, and waist circumference [26]. 

In the Multi-Ethnic Study of Atherosclerosis cohort, when compared to those with lower total tree nut consumption (never/rarely), a higher consumption (≥5 times/week; portion size not shown) was associated with lower levels of inflammatory biomarker CRP (1.98 vs. 1.69 mg/L). For peanuts and peanut butter, a higher level of consumption (≥5 times/week) compared to never/rarely consumed was associated with lower IL-6 (1.11 vs. 1.24 pg/mL) [27]. The same cohort also investigated the influence of anthropometric measures (BMI and waist-to-hip ratio (WHR)) and ethnicity on the relationship between total nuts and seed consumption and inflammatory markers. Lower levels of CRP and IL-6 and WHR < 0.94 cm were observed in groups with higher nut and seed consumption, while individuals with BMI ≥ 28 kg/m^2^ and WHR ≥ 0.94 cm had lower fibrinogen values. Regarding ethnicity, lower CRP, IL-6, and fibrinogen levels were found in the Caucasian population with higher total nut and seed consumption. 

Cross-sectional data from the NHS and HPFS demonstrated that higher nut consumption (≥5 times/week; portion size—28 g/day) was associated with lower CRP (OR = 0.84; 95% CI = 0.74–0.95; *p* trend = 0.006) and IL-6 (OR = 0.88; 95% = 0.79–0.99; *p*-trend = 0.016) [27], but not TNFR-2 [29]. In addition, the Moli-Sani Study cohort [28] with a follow-up of 4.3 years showed that individuals with higher nut consumption (≥8 times/month; portion size not shown) had lower levels of low-grade inflammation (CRP, platelet count, and neutrophil-to-lymphocyte ratio). Higher nut consumption was also associated with all-cause and cancer mortality when inflammatory markers were combined with other variables. Compared with those who reported never consuming nuts, participants with regular nut consumption had a 34% lower risk of all-cause mortality and a 36% reduction in cancer mortality. 

In summary, although tree nuts and peanuts are rich in antioxidants and other bioactive components, cohort studies have not consistently considered the association between oxidative stress and inflammatory markers and the risk of fatal and non-fatal disease outcomes when diet evaluations occurred at baseline. Thus, we rely on evidence from RCTs (albeit inconclusive currently) to clarify the relationship between nut consumption and oxidative stress and inflammation. 

### 2.2. Evidence from RCTs

A few systematic and narrative reviews and meta-analyses have summarized findings from RCTs on the role of nut consumption (almonds, Brazil nuts, hazel nuts, pistachios, walnuts) on inflammatory biomarkers [30,31,32,33,34,35,36]. There is some evidence, although inconsistent, that nuts may ameliorate inflammation. 

When considering inflammatory markers, a meta-analysis on almond consumption [32] mainly noted a significant decrease in serum CRP. This effect was observed more consistently among adults free of obesity and generally healthy than those with metabolic disorders [37,38,39], and when the intervention was at least 12 weeks. Among other inflammatory cytokines assessed, almond consumption seemed to lower IL-6 significantly in some [40,41], but not all studies [38,42], with no significant changes in TNF-α or endothelial adhesion molecules [32]. The decrease in serum IL-6 attenuated when adjusted for weight loss [42]. Whether almond consumption affects adhesion molecules is unknown, since these markers have not been measured in studies conducted thus far. The evidence appears to primarily suggest a modest lowering of serum CRP with almond intake [32]. However, it is critical to highlight that CRP levels may be associated with numerous factors, including infections, trauma, and non-dietary lifestyle factors [43]. Consequently, researchers should assess multiple inflammatory parameters and adjust for potential confounding variables. 

In contrast, a meta-analysis of nine RCTs [33] on walnut intake on CRP that ranged in duration from four weeks to twelve months, with comparators varying from protein foods (eggs, meat), dietary fat (olive oil) to a walnut-free habitual diet, revealed only a non-significant change. The effects of walnuts on other inflammatory cytokines such as IL-6, TNF-α, and IL-1β are inconsistent, mainly tending towards a null effect [33], except when the duration exceeded 24 weeks [44]. Regarding adhesion molecules, although the meta-analyses concluded no significant effect on walnuts (331), individual RCTs suggest a somewhat favorable effect on adhesion molecules [45,46,47]. The discrepancies in the findings may be partly attributed to the small sample size and short duration of most of these RCTs [33]. Meta-analyses performed with a small number of studies that vary in study design, duration, and subject characteristics, and are limited in sample size, may introduce sampling error that leads to bias in reporting [48]. A key consideration is the intensity and duration of the intervention, which was two years in the Walnuts and Healthy Aging (WAHA) study [44]. This significantly exceeded even the longest trial of 24 weeks cited in the meta-analysis on walnuts [33], highlighting the relevancy of the exposure period. In the WAHA study, the daily ingestion of walnuts over two years in healthy older adults significantly reduced several inflammatory biomarkers including E-Selectin, IL-6, TNF-α, granulocyte-macrophage colony stimulating factor, interferon-γ and, most strikingly, IL-1β (which are associated with CHD). 

In a recent systematic review of hazelnut consumption on cardiometabolic risk factors [36], only a few studies assessed the outcomes of inflammation and/or oxidative stress markers and revealed null effects. The seemingly favorable effect on CRP and VCAM-1 was from a study that utilized a less rigorous sequential or single intervention design, and/or combined hazelnuts with cocoa as the intervention [49]. Similarly, for oxidative stress markers, oxLDL decreased and antioxidant enzyme gene expression increased, but only post-prandially [50] or was noted only in uncontrolled studies [36]. The impact of other tree nuts (Brazil nuts, cashews, macadamias, pecans, pine nuts, pistachios) and peanuts on inflammatory biomarkers has been explored less [30,31,34,35]. However, many of these nuts are rich in phytochemicals and other anti-inflammatory compounds similar to walnuts and almonds [16] and warrant further exploration. 

Collectively, the evidence on the effect of nuts and inflammation indicates a significant gap in research. Overall, there is a trend towards modest reductions in CRP and IL-6 with almond intake, although this is attenuated when adjusted for weight loss for IL-6 [32]. Short-term studies (≤24 weeks) with walnuts mostly suggest a significant reduction in endothelial adhesion molecules [33]. For other tree nuts and peanuts, conclusions cannot be drawn due to either insufficient data or inconclusive evidence. However, these inconsistencies and seeming null effects on inflammatory biomarkers may be explained by the study designs implemented, since favorable changes on multiple inflammatory biomarkers were noted when the exposure to a single nut was longer, as was seen with walnuts when consumed for two years [44]. 

A systematic review of 16 RCTs showed an overall favorable effect of nuts on oxidative stress markers [17,51,52]. However, the strength of the evidence is weak, due mostly to the variations in the markers assessed. A modest beneficial effect was specifically observed for almonds, Brazil nuts and mixed nuts. Almonds at doses ranging from 37 g/d to 74 g/d for at least four weeks consistently lowered oxLDL levels, especially in adults with hyperlipidemia and T2D [53], but other lipid peroxidation products such as MDA, or antioxidant capacity, were not responsive to almond intervention. In a recent meta-analysis on Brazil nuts [35], five RCTs that examined the effect on antioxidant enzyme activity noted a positive effect on the selenium-containing glutathione peroxidase activity (GPx) and the increased expression of Nrf2 [54,55]. Brazil nuts have high selenium content [16,35], which is reflected in high serum selenium levels following the intake of just one Brazil nut a day [54]. Another significant marker of oxidative stress, 8-OHdG, reflecting DNA damage, was reduced as a result of Brazil nut intervention in hemodialysis patients [56], indicating that individuals with this disease state may have amplified oxidative stress. For walnuts specifically, there seems to be a positive impact on oxidative stress markers such as oxLDL and antioxidant capacity, but only postprandially following the ingestion of a walnut meal [57]. In the short term (≤12 weeks), walnuts had no effect on oxLDL [51] unless provided along with almonds and hazelnuts, in which case they lowered the urinary excretion of 8-OHdG following a 12-week intervention in those with metabolic syndrome [58]. For other tree nuts (cashew, pecan, pistachio) and peanuts there are mostly null findings concerning oxidative stress markers, largely because ≤ 2 RCTs have evaluated these markers for these nut types [51]. 

An increase in one or more of the antioxidant enzymes has been noted for some nuts [51], although mostly among adults with metabolic conditions accompanied by a heightened state of oxidative stress [53,56]. Dose and duration may be more important than the type of nuts, since studies of 8 weeks or longer, and at least 40–60 g/d (except for Brazil nuts, with just 1 unit/d) seem to have some clinically relevant impacts on antioxidant enzymes. A less common assay for antioxidant capacity is the ferric-reducing antioxidant property (FRAP), which has been used in a few studies and is increased with nut intervention [57]. Different forms of a nut, specifically walnuts, (whole, skin, oil, defatted) improve FRAP acutely [59]. However, the defatted walnuts have the least effect, suggesting that some of the polyphenol antioxidants may be removed during the fat removal, lowering the antioxidant potential. Inconsistencies across studies on the choice of biomarkers or analytical processes used to measure antioxidant capacity and oxidative stress preclude drawing any conclusions. 

The evidence from the various meta-analyses and systematic reviews discussed in this section, and the authors’ interpretations of the current knowledge and gaps in research, are summarized in Table 2. The discrepant findings for most nut types on oxidative stress markers illustrate several shortcomings in these studies, including small sample size, short duration of the intervention (mostly ≤ 8 weeks), varied nut dose (<10 g/d to >100 g/d), form of the nut (whole nut, nut butter, nut oil), participant characteristics (age, smoking status, health status e.g., healthy, presence of diabetes, metabolic disorders, high CVD risk), and the control diet choice (habitual nut free diet, specific high fat or high protein food, low-fat or MedDiet). While there are several limitations, the most significant current gap in knowledge is the lack of assessment of a more robust profile of oxidative stress biomarkers. 

## 3. Nuts: Antioxidant and Anti-Inflammatory Mechanisms

Inflammation and oxidative stress, two of the common mediators of NCDs, result from a dysregulation caused by an increased production of reactive oxygen species (due to an imbalance in oxidative and antioxidative pathways in the cell), producing pro-inflammatory cytokines [2,8]. These processes are regulated by two transcriptional factors, NFκβ and Nrf2, the former induced when oxidative stress is high and resulting in a pro-inflammatory state, and the latter responsible for mitigating the state of inflammation and oxidative stress. The plethora of bioactive compounds in nuts, such as unsaturated fatty acids, tocopherol, selenium, copper, fiber, phytosterols, polyphenols, and other phytonutrients, independently and synergistically modulate inflammation and oxidative stress by influencing one or more of the pathways involving these two nuclear factors (Figure 1). 

With respect to oxidation, PUFAs act as a pro-oxidant whereas MUFAs are mostly neutral or do not contribute to oxidative stress. Nuts predominantly have MUFAs, except walnuts, which are high in PUFAs. Studies of nuts lowering oxLDL have mostly been observed with hazelnuts, almonds, and Brazil nuts, but not walnuts [51]. Importantly, no adverse effects have been reported, possibly due to the presence of other powerful antioxidants that might counteract the oxidant potential of PUFAs [16,17]. Some of the phytochemicals in nuts (phytosterols, and polyphenols), as well as selenium, can up-regulate the Nrf2 pathway which stimulates the antioxidant response element (ARE) gene transcription and the various antioxidant enzymes it encodes, including GPx, superoxide dismutase and catalase [60]. When Nrf2 is activated, it directly counteracts the NFκβ pathway, and reduces the pro-inflammatory state [60,61]. Antioxidants such as γ-tocopherol and phytonutrients directly inhibit the NFκβ pathway and suppress the pro-inflammatory state [62]. Unsaturated fatty acids, particularly ALA found in walnuts, may exhibit anti-inflammatory activity, potentially via the modulation of cyclooxygenase and lipoxygenase pathways [63]. Vitamin E (tocopherols), polyphenols, phytosterols and selenium are powerful antioxidants that are mostly responsible for the antioxidant capacity of tree nuts and peanuts [16,17,18]. Selenium and copper, notedly high in some nuts (such as Brazil nuts), are cofactors of antioxidant enzymes, including GPx that suppresses oxidative stress [58]. Overall, the bioactive compounds in the nut matrix, both independently and synergistically, may be exerting antioxidant and anti-inflammatory effects. 

## 4. Scope for the Future

Routine tree nut and peanut consumption improves health and reduces the risk of NCDs through multiple pathways, including a reduction in inflammation and oxidative stress [6,7,12,13,14,15]. Although the relationship between inflammation and oxidative stress is well recognized, cohort studies conducted to date have only evaluated the association between nut intake and inflammatory biomarkers, not oxidative markers, or their association with disease outcomes. Thus, future cohort studies could consider both oxidative stress and inflammatory markers as intermediate mediators between exposure and disease outcomes. Furthermore, cohort studies should include repeated measures of diet during the follow-up to reduce bias associated with changes that may be made in lifestyle behaviors over time. 

The comprehensive evidence from RCTs on the role of nuts on inflammation and oxidative stress biomarkers remains unresolved, although there is evidence of beneficial effects for some nuts such as almonds and walnuts on select markers of inflammation, and for Brazil nuts on oxidative stress. Historically, RCTs have considered inflammation and oxidative stress biomarkers as secondary outcomes [30,31]. However, in nut intervention trials, the CVD risk reduction achieved was greater than predicted only by the LDL-C lowering [19]. This indicates that other mechanisms could contribute to the overall CVD risk reduction, which perhaps encouraged the evaluation of inflammation and oxidative biomarkers to explain this gap. These studies were likely underpowered to assess these biomarkers, as they were not a prespecified primary outcome. Well-designed RCTs with a large sample size, evaluating inflammation and oxidative stress biomarkers as primary outcomes, are vital to clarify some of the inconsistencies that exist at present. These RCTs should consider the duration (≥24 weeks), dose (40–60 g/d), how nuts are incorporated (added to the habitual diet, substitution for nutrient/food or displacement), the comparator diet (habitual nut-free diet or high carbohydrate snack, or other fat source), potential confounders (including age, sex, genotype and lifestyle factors), and the participants’ health status (healthy, high risk for T2D or CVD, obesity). 

One of the major confounders in studies assessing the association between nut intake and inflammation and oxidative stress biomarkers is body weight. While most studies adjust for weight or BMI, the exposure’s effect may differ among those with and without obesity [30,31,32,33,34,35,36]. In addition, studies show that nuts, when part of a low-calorie diet (LCD), may produce similar weight loss compared to a nut-free LCD [64]; however, nut-enriched LCD may have additional benefits, as in favorably modifying inflammatory biomarkers. This implies that nuts may influence inflammatory biomarkers independent of body weight, but future studies have to untangle the complex connections between body weight, inflammation and nut consumption. 

The biggest gap in this area of nut research is the apparent lack of evaluation of multiple markers of inflammation and oxidative stress. No single biomarker is ideal, as each marker may be associated with different metabolic conditions (high CRP associated with CVD, for example) or originate from different processes (8-OHdG for DNA damage, F2 isoprostanes for lipid peroxidation). The current consensus summarized in Table 2 is that for some nuts there are few to no RCTs that have evaluated these outcomes. For others, comparing outcomes from different studies is challenging, since they have not always measured the same markers, or have used different study designs, sometimes of less rigor [30,31,32,33,34,35,36]. In the WAHA study, one of the strengths besides the long duration (two years) of exposure to walnuts was the inclusion of ten inflammatory biomarkers [44], where six showed a favorable modification with walnut consumption. There is also a concern that there may be large inter- and intra-individual variability for these biomarkers, that would make it difficult to detect any real change caused by diet modification [65]—this makes a case for including a large sample size. The validity of measuring these markers only in a fasting state, which is currently the practice [30,31,32,33,34,35,36], is considered insensitive, especially in healthy individuals. Instead, the use of inflammatory challenges has been proposed [66] and much work is needed in this area before considering for future studies on diet and inflammation/oxidative stress. In addition, metabolomic signatures that better represent low-grade inflammation could be identified and may be more useful than single markers.

Along with assessing multiple biomarkers, it would be useful to explore other mechanisms by which nuts influence inflammation and oxidative stress. Genetics, epigenetics, and omics would be relevant considerations for the future [67]. We have limited evidence that Brazil nuts increase the gene expression of antioxidant enzymes and suppress the NFκβ pathway [55], and that pistachios lower IL-6 gene expression [68]. This needs to be verified with more well-designed studies that include other nut types. Epigenetic changes can also modify inflammatory genes, as demonstrated in a cohort study of high CVD-risk patients following a MedDiet with mixed nuts [67]. While DNA methylation assays are expensive, they may be well worth the investment as plant food bioactives seem to favorably modify inflammation and oxidative stress through DNA methylation changes [69]. Finally, the connection between the gut microbiome, whole-body inflammation, and oxidative stress cannot be ignored. Given the beneficial role of nuts on the gut microbiome [70], future studies should include the inter-relationships between the gut axis and immune system in nut exposure studies. 

In secondary prevention trials of CVD and type-2 diabetes, new frontiers to explore would include inflammation and oxidative stress. One would expect patients with NCDs to have elevated inflammation, and thus have a better response to an intervention. Although a small study in adults with CHD failed to show any change in inflammation with 30 g/d of pecans [71], another study showed that a mixed nuts or extra virgin olive oil enriched MedDiet was associated with increased atheroma plaque stability and reduced vascular inflammation compared to a low-fat MedDiet [72]. Thus, in healthy individuals with normal values of these biomarkers it may be challenging to see the intervention effect, and hence choosing individuals with elevated inflammation and oxidative stress as an inclusion criterion may be a better option.

Cardiometabolic disease is often associated with non-alcoholic fatty liver disease (NAFLD), with oxidative stress and inflammation considered mediators of this condition [2]. Preliminary evidence suggests that frequent nut consumption (≥1 time/week) is inversely associated with NAFLD, at least in men [73]. Additionally, other polyphenol-rich plant foods (green tea, fruits, and spices) have been shown to protect against fatty liver [74]. With NAFLD becoming a significant public health problem, and given that nuts have abundant polyphenols and other anti-inflammatory nutrients, a critical next step would be to determine if nut intake could lower liver fat fraction in those with NAFLD. Alongside the nut type, duration and dose, such studies must carefully consider the background diet, include nuts in the context of LCDs, and use accurate techniques such as magnetic resonance imaging. Alongside NAFLD, future research can build on preliminary work highlighting a beneficial role for nuts in inflammatory/oxidative stress indices in women with polycystic ovary syndrome (PCOS) and bone health [75,76]. Another emerging trend since the COVID-19 pandemic has been research focused on identifying plant foods to boost the immune system. Oxidative stress and inflammation are potent modulators of the immune response [77] and, thus, evaluating the role of nuts in bolstering the immune response would be timely and critical. 

Shifting our approach from utilizing biomarkers to assessing inflammation and oxidative stress, future studies could consider a dietary approach such as the dietary inflammatory index (DII). In the absence of a significant decrease in serum inflammatory markers, a lowering of the DII score may be a surrogate that implies an anti-inflammatory effect. In a six-month dietary intervention study (MedDiet versus low-fat control), a decrease in DII scores was observed with the MedDiet [78], pointing to the anti-inflammatory potential of the MedDiet. Studies exploring the DII of a diet with a single food (nut) intervention may thus be valuable to the overall understanding of the role of nuts in lowering inflammation. Moreover, higher DII scores seem to correlate with CVD and related adverse clinical events [79]. 

## 5. Conclusions

In conclusion, the current evidence from cohort studies and randomized clinical trials suggest that tree nuts and peanuts packed with potent bioactive nutrients (MUFAs, PUFAs, vitamin E, selenium and copper) and non-nutrients (fiber, polyphenols and phytosterols) have the potential to reduce inflammation and oxidative stress. However, the evidence is only modest for some, inconsistent for few, and has not been evaluated for many nut types. This creates excellent opportunities for future research to focus on well-designed RCTs that consider many of the limitations described in this narrative review, to further our understanding of the role of nuts in reducing inflammation and oxidative stress. A strong consensus is that including nuts in the habitual diet can help mitigate the risk of several chronic diseases. However, the evidence base for nuts in this area must be expanded to promote food (nut)-based strategies, to lower inflammation and oxidative stress for precision and public health nutrition. 

## Figures and Tables

**Table 1:** Cohort Studies: Associations between nuts and inflammatory biomarkers.

Reference	Study Country	Sample Size Gender	Follow-Up	Nut Type Amount	Outcome	Inflammatory Biomarkers
Li et al., 2009 [25]	NHS, USA	6.309 Female	1989–1990	Peanuts and mixed nuts Almost never or ≥5 servings/week (Portion size—28 g/day for nuts and 16 g/day for peanut butter)	Incident CVD	TNFR-2, ICAM-1, E-selectin, CRP, and fibrinogen (No changes)
Jiang et al., 2006 [27]	MESA, USA	6.080 Female, Male	Baseline	Mixed nuts, seeds, or peanuts/peanut butter Never/rare or ≥5 times/week (Portion size—no data)	Inflammation biomarkers	↓CRP, ↓IL-6 and ↓fibrinogen
Mantzoros et al., 2006 [26]	NHS, USA	987 Female	1989–1990	Mixed nuts Quintile of nuts intake (Portion size—no data)	Adipocytokine	↑Adiponectin
Bonaccio et al., 2015 [28]	Moli-sani Study, Italy	19,386 Female, Male	4.3 years	Walnuts, hazelnuts, almonds, and peanuts Never or ≥8 times/month (Portion size—no data)	Total and specific mortality	↓CRP, ↓platelet count and ↓neutrophil to lymphocyte ratio
Yu et al., 2016 [29]	NHS HPFS, USA	NHS (3654) HPFS (1359) Female, Male	NHS (1989–1990) HPFS (1993–1995)	Peanuts, mixed nuts, and peanut butter Almost never or ≥5 times/week (Portion size—28 g/day)	Inflammatory biomarkers	↓TNFR-2, ↓CRP and ↓IL-6

NHS—Nurses’ Health Study; MESA—Multiethnic study of atherosclerosis; HPFS—Health professional follow-up study; CVD—cardiovascular disease; TNFR—Tumor necrosis factor receptor; CRP—*C*-reactive protein; IL-6—Interleukin 6. ↓—higher consumption of nuts associated with lower levels of the inflammatory markers; ↑—higher consumption of nuts associated with higher levels of the anti-inflammatory marker.

**Table 2:** Nuts and inflammation and oxidative stress biomarkers—current evidence from randomized controlled trials and research gaps.

Nut Type Reference [#]	CRP	IL-6	TNF-α	Adhesion Molecules	OxLDL	Antioxidant Enzymes	Oxidized Metabolites
Almonds [32,51]	↓	↓	↔	↔	↓	-	↔
Brazil nuts [35,51]	↔	-	-	-	↔	↑	↔
Hazelnuts [36,51,52]	↔	-	-	-	-	-	-
Pistachios [34,51]	↔	-	-	-	↔	-	-
Walnuts * [33,44,51,52]	↔	↓	↓	↓	↔	-	↔
Other ** [30,31,51]	-	-	-	-	-	-	-

Adhesion molecules (E-selectin, ICAM-1, VCAM-1); antioxidant enzymes include glutathione peroxidase or catalase, superoxide dismutase activity or gene expression; oxidized metabolites include 8-hydroxy deoxy guanine, urinary isoprostane, MDA. ↑ Significant increase; ↓ Significant decrease; ↔ Null impact or conflicting findings; - Not sufficient evidence (i.e., gaps in research) * Walnuts affect inflammatory biomarkers while mostly null according to a meta-analysis [33]; exposure to walnuts for two years produced a more robust anti-inflammatory effect [44], which is reflected in this table. ** Other tree nuts (cashews, macadamia, pecans, pine nuts) and peanuts.
